# The Utility of Grimace Scales for Practical Pain Assessment in Laboratory Animals

**DOI:** 10.3390/ani10101838

**Published:** 2020-10-09

**Authors:** Daniel Mota-Rojas, Adriana Olmos-Hernández, Antonio Verduzco-Mendoza, Elein Hernández, Julio Martínez-Burnes, Alexandra L. Whittaker

**Affiliations:** 1 Neurophysiology, Behavior and Animal Welfare Assessment, DPAA, Universidad Autónoma Metropolitana, Xochimilco Campus, Ciudad de México 04960, CDMX, Mexico; dmota100@yahoo.com.mx; 2 Division of Biotechnology—Bioterio and Experimental Surgery, Instituto Nacional de Rehabilitación-Luis Guillermo Ibarra Ibarra (INR-LGII), Tlalpan 14389, CDMX, Mexico; adrianaolmos05@yahoo.com.mx (A.O.-H.); cnrverduzco@hotmail.com (A.V.-M.); 3 Department of Clinical Studies and Surgery, Facultad de Estudios Superiores Cuautiltán UNAM, Cuautitlán Izcalli 54714, Estado de México, Mexico; elein_ht@comunidad.unam.mx; 4 Graduate and Research Department, Facultad de Medicina Veterinaria y Zootecnia, Universidad Autónoma de Tamaulipas, Cd Victoria 87000, Tamaulipas, Mexico; jmburnes@docentes.uat.edu.mx; 5 School of Animal and Veterinary Sciences, University of Adelaide, Roseworthy Campus, SA 5116, Australia

**Keywords:** facial expressions, pain, grimace scales, mice, rat, rabbit

## Abstract

**Simple Summary:**

Grimace scales for laboratory animals were first reported ten years ago. Yet, despite their promise as pain assessment tools it appears that they have not been implemented widely in animal research establishments for clinical pain assessment. We discuss potential reasons for this based on the knowledge gained to date on their use and suggest avenues for further research, which might improve uptake of their use in laboratory animal medicine.

**Abstract:**

Animals’ facial expressions are widely used as a readout for emotion. Scientific interest in the facial expressions of laboratory animals has centered primarily on negative experiences, such as pain, experienced as a result of scientific research procedures. Recent attempts to standardize evaluation of facial expressions associated with pain in laboratory animals has culminated in the development of “grimace scales”. The prevention or relief of pain in laboratory animals is a fundamental requirement for in vivo research to satisfy community expectations. However, to date it appears that the grimace scales have not seen widespread implementation as clinical pain assessment techniques in biomedical research. In this review, we discuss some of the barriers to implementation of the scales in clinical laboratory animal medicine, progress made in automation of collection, and suggest avenues for future research.

## 1. Introduction

Animal welfare is an important societal concern [1,2]. The use of animals in biomedical scientific research is widespread, and globally significant, with approximately 115 million animals used per year [3]. Incontrovertibly, there is an ethical obligation to safeguard welfare of these animals through employing strategies to minimize pain, fear, and distress [4,5,6], in addition to the promotion of positive welfare states. However, to achieve this, validated methods for identification of animal emotional state are required. Despite significant research attention, ascertaining nature and strength of animal emotion remains a challenging task [7,8,9,10,11].

The study of emotion in laboratory animals has typically focused on aversive states such as pain. This area of study was driven by two perspectives: a scientific and welfare standpoint. The scientific viewpoint, based on the extrinsic value of the animal, relates to the robustness of results acquired from animal models. There is an abundance of data on the impact of pain on a wide range of metabolic, immunologic, and other processes in the body. These alterations introduce variability or confound interpretation of results [12,13,14]. The welfare viewpoint, considering the intrinsic value of the animal, assumes that pain occurs frequently in animal models and should therefore be avoided or minimized for the benefit of the animal. Notwithstanding, differences between these viewpoints in terms of underlying motivation for study, the requirement for a reliable, practical method for assessment of pain is shared by both.

Recently, evaluation of complex motor responses, such as facial and corporal expression was proposed as a neurobiological readout of mammalian brain neuro-circuitry associated with emotional experience [11,15,16,17]. The former has received significant research attention, especially in rodents, as a potential assessment method for both positive and negative emotional states [9]. There remains controversy as to the communicative function of facial expressions in rodents, since these species tend to prioritize other senses such as olfaction and touch in communication [8]. However, the finding that in mice, lesions of the insular cortex, modulate facial pain expressions supports the use of facial expression assessment. The insular cortex is associated with human pain perception; hence it is assumed by analogy that facial grimace may represent a negative emotional experience [18]. Furthermore, studies on empathy tends to suggest that rodents are communicating the presence of a painful state to others, to elicit an empathic response [19]. Although not specifically demonstrated, it is feasible that this may be occurring through interpretation of facial expression [8]. Additionally, it was recently shown through the use of machine learning methods that facial expressions in mice may not only indicate direction of effect or valence of emotion (positive or negative), but intensity and persistence [20].

Attempts to standardize evaluation of facial expressions for pain assessment has culminated in the development of the “grimace scales”. These were developed originally for mice [18] and were adapted for use in rats [21], rabbits [22,23], sheep [24,25], ferrets [26], cats [27] and horses [28]. Grimace scales are simplified methods for evaluating facial expressions specifically related to pain based on the assessment of action units focusing on the eyes, ears, and cheeks. The utility of the scales was well-established across a range of laboratory animal species and animal model types. However, this evaluation has typically focused on their use via retrospective video recording review, and as a research tool to obtain data relevant to the animal model. There are fewer dedicated studies into the scales as ‘bedside’ pain assessment tools for rapid evaluation of pain status in laboratory animals in order to implement humane endpoints or provide analgesia. Therefore, the focus of this review is to discuss the practical utility of grimace scales in a range of laboratory animal species, identifying barriers to their use and potential confounders. The focus will be on laboratory animal rodents as the most common species used in biomedical research, but research from other species will be drawn upon. It is anticipated that this review will guide biomedical researchers, animal technicians and ethics committees when implementing pain assessment methods as part of research protocols.

## 2. History of Facial Expression Scoring for Pain in Laboratory Animals

In recognition of the poor translation of outcomes from animal pre-clinical studies on pain physiology and analgesic development to humans [29,30], there has been a recent focus on development of methods for assessment of the affective pain response using non-evoked (spontaneous) responses [31]. Grimace scales are one such response derived from human facial codification scales [32,33]. The Facial Action Coding System (FACS) systematically catalogs all possible movements of the facial muscles, or combinations of them, such as lowering the eyebrows, tightening and closing the eyelids, wrinkling the nose, and raising the upper lip. Categorization of changes in these muscle movements or so-called “Facial Action Units” (FAU) enables facial recognition and categorization of emotions [16,34]. The finding that facial codification scales could quantify pain in humans with limited or non-existent verbal communication [35], provided the basis for using FAU in the development of grimace scales (GS) for animals (see [36]).

The mouse grimace scale (MGS) was the first to be developed. Langford et al. [18] in 2010 applied a nociceptive abdominal constriction test through administration of acetic acid that allowed the elucidation of facial action units that reliably detected pain. Validation was performed using a variety of traditional preclinical pain assays [18]. Five action units were described: (1) orbital tightening, (2) nose bulge, (3) cheek bulge, (4) ear position and (5) whisker change. A year later, Sotocinal et al. [21] in 2011 published the rat grimace scale (RGS) comprising four action units, due to consolidation of nose and cheek flattening into one unit. Utility of the RGS to detect pain was demonstrated in standard pre-clinical nociceptive tests in addition to following a surgical laparotomy procedure. Furthermore, the RGS was shown to be modified after analgesic administration indicating the specificity to pain [21]. Furthermore, the development of grimace scales in other common laboratory animal species followed, see Table 1.

## 3. Terminology Around Pain Classification and Assessment

A variety of terms are often used to describe pain and the assessment methods applied to it. Pain is usually classified according to the duration of its effect or its originating source within the body [39,40]. Acute pain arises at the time of injury and is often experienced as different in nature to the alternatively described ‘chronic’ pain. The latter generally referring to pain experienced over a longer duration, although there appears to be no accepted duration marking the transition from acute to chronic pain [41]. An alternative distinction between the two time-course descriptors was suggested by scientists: that related to functionality. Acute pain is argued to be adaptive, provoking a learned response by the animal to avoid a similar painful insult in the future [39]. Chronic pain on the other hand is said to be maladaptive [42]. However, this latter point is controversial with a variety of studies (see [43] for review) suggesting that pain-related hypervigilance may influence estimation of risk, subsequent behavior, and thus enhance survival.

Pain scales themselves are often described in terms of their validity, reliability, sensitivity [41]. Validity describes the extent to which the scale measures its intended outcome i.e., pain. There are several sub-categories describing validity. The most commonly referred to in the context of grimace scales are face validity and construct validity. Face validity describes what the test appears to be measuring i.e., pain. Construct validity relates to the extent to which the scales measure that specific construct. Therefore, the test needs to be both sensitive and specific to pain [44,45]. In pain studies construct validity is often determined using an applied analgesic test, since this is assumed to reduce pain and thereby reduce grimace scores if the test is truly pain-related [44]. External validity refers to how generalizable the measure is to other settings. In the context of grimace scales this is relevant in taking the scales from research scenarios to the clinical setting. This relates to practicability to perform during the working day, simplicity of the task, as well as the need for equipment and training. To date, this is the area that has received the least attention with regard to grimace scales.

Reliability refers to the scale producing the same result each time it is used both within, and between animals, and time points [46]. In the context of grimace scales, this is determined by the variability resulting in a single observer’s measurements (intra-observer variability), the variation between different observers’ measurements (inter-observer), and variability between laboratories or research centers [44]. Sensitivity describes the ability of the scale to accurately identify changes in the degree of pain such that subtle changes are recognized [45]. In the context of pain scales this is often indicated when scale changes that occur correlate in direction, and proportion with other measures [45]. It is common in assessment of pain in veterinary species to achieve measurement accuracy in pain scoring by using a smaller number of broad category groups, such as mild, moderate, and severe, rather than expecting sensitivity when small differences in scores are considered. The following will consider how all of these measurement characteristics may influence the clinical applicability of grimace scales for use in biomedical research.

## 4. Clinical Applicability of Grimace Scales in Biomedical Research

### 4.1. Development of Real-Time Grimace Scores

There is now an extensive body of literature on the application of grimace scales in a range of animal models used commonly in biomedical research. The majority of this validation work has occurred in rodent models. It is beyond the scope of this review to describe all of the models used but the range includes oncology (see e.g., [47,48,49,50]), infectious disease [51], pain models [48,52,53], neurological conditions [33,54,55], genetic conditions [56], and maxillofacial interventions [49,50,57]. However, the vast majority of research to date has performed grimace scoring retrospectively from captured video footage.

Retrospective scoring is likely superior when using grimace scores to inform research outcomes, for example determining efficacy of analgesics or success of model induction. These methods allow for the possibility of replication, by multiple observers where appropriate, with an increased time available for scoring at the researcher’s leisure. A cage-side or ‘real-time’ method on the other hand would ideally provide instant assessment allowing interventions to support welfare, for example by implementing humane endpoints or administering analgesics. Development of the latter is clearly of more interest to ethical review committees and animal carers needing to make rapid clinical decisions. To date there has been substantially less focus on development and validation of real-time methods.

Miller and Leach [58] in 2015 performed the first comprehensive evaluation of a real-time method applied in mice. In this study, both retrospective and real-time scoring were compared. Real-time scoring was performed by observing mice three times over a 10 min period, while animals were being filmed for the retrospective analysis. Grimace scores were calculated by summation of each action unit as described by Langford et al. [18], and totals were then averaged across the observation points. Live scores were always found to be significantly lower than corresponding retrospective video scoring. The authors posed that this could have resulted from the activity levels and changing nature of the face during live scoring. Blinking for instance, resulting in a score of 0 for orbital tightening, will likely be selected at least some of the time as a result of random chance selection of photographs for scoring. In a real-time scenario, the rapid nature of blinking will likely preclude its scoring. Similarly, Chartier et al. [47] in 2020 also found consistently lower scores from live scoring compared to retrospective scoring in a mouse model of colitis-associated colo-rectal cancer. One potential explanation for this trend is that the presence of a human observer influences performance of the facial action units, for example, an increased alertness to the human (predator) could lead to wider eyes and ‘pricked’ ears, lowering the grimace score. On the contrary, intriguing findings from Sorge et al. [59] demonstrated that not all observers are equal, with no impact of a female observer on scores in rats and mice (obtained retrospectively), but a reduction of scores in the presence of a male [59]. In the first investigation of real-time scoring in rats, Leung et al. [60] in 2016 found that interval observations (15 s of observation) were able to discriminate between control and analgesic–treated groups whereas point observations (conducted several times over a period) showed poor group discrimination. In this study, substantial variability was seen between single observations of either point or interval. Limits of agreement, with a retrospective scoring system were however fairly large with a 0.5 score range either side of the bias meaning there is was a substantial risk of both over or underestimating the score. Furthermore, point scoring became generally unreliable at discriminating groups when done for less than 2 min, assumed to be due to a loss of power due to fewer observations. A later rat study by the same research group [61], investigated the interval method compared to a retrospective method in a colitis model showing the former to be reliable in predicting pain, with scores similar to the standard method.

The implications of these findings for clinical pain assessment are several. Firstly, it needs to be considered that although good discriminant ability was generally found in these studies, results were obtained by statistical combination of multiple scores. In a clinical scenario, an observer is likely to take one score, and not have the means or time to mathematically manipulate the values to arrive at a reliable score. Secondly, the Leung et al. [60] study suggests that variability across the observation period is likely and that at least 2 min of observation is needed. It is unlikely to be practical for a caregiver to spend 2 min per animal performing pain assessment across a study. In this case, some other more general method of distress measurement is likely to be needed to ‘triage’ animals for secondary grimace assessment. There has been no investigation of the effect of movement to the clear cages, in isolation, as typically occurs in grimace studies as opposed to scoring occurring in the home cage environment. Several factors may influence the grimace scoring between these two scenarios. The novelty of the scoring box may trigger a state of alert influencing grimace scores in a similar vein to that suggested for the presence of a human observer. This novelty may indeed contribute to the variability seen between scores over time since habituation will eventually occur. Alternately, if scoring in the home cage, the presence of cage furniture, a potential more relaxed state of the animal in its familiar environment, or even the influence of circadian rhythms (see later) may all variously influence the action units or ability to see them accurately. A further consideration with real-time scoring is that there may be an inherent observer bias as the animal’s overall demeanor, or presence of other pain behaviors such as twitching may be noted leading the observer to err on the side of higher action unit scores when unsure. This is not necessarily an issue per se in a clinical scenario since the goal is to recognize sick animals for further evaluation and treatment. However, these other behaviors may not be unique to pain but represent general sickness behavior that may not be able to be rectified by analgesic administration, and hence inappropriate medication administration may occur. If such biasing were occurring it would be expected that there may be differences in grimace scoring between observers experienced with working with the species in question versus more naïve observers [47].

Notwithstanding, these findings some research groups do appear to have been able to use the MGS or RGS in a point observation, real-time scenario to obtain predicted results. For example, in chemotherapy-induced toxicity models in mice [62], and rats [63] single grimace scores allowed distinguishing between groups and followed the progression of the disease course as expected, after induction of chemotherapy-induced gut toxicity. Alternately, Hsi et al. [64] in 2020 were unable to use point mouse grimace scores to distinguish between groups either supplemented or not with dextrose following bariatric surgery. However, in this experimental design there was no sham group so it is unknown whether the MGS can reliably determine pain in this model [64].

There is clearly a need for further validation of real-time observation methods with a particular focus on one-off observations versus a series of observations, correlation with other established measures of pain assessment, inter-observer variability and home cage versus novel area.

### 4.2. Impact of Biology and the Environment

#### 4.2.1. Strain and Sex Differences

There is some evidence that features of biology, performance of routine procedures, or aspects of the environment may influence grimace scores. This has implications for setting of intervention scores (see later), and should be a consideration in driving further research or recommendations for application to clinical practice.

Aspects of biology have perhaps been the most researched with regard to their impact on grimace scores. The greatest implication of such changes likely relates to any differences between rodent strains or stocks given the wide range typically used in research. In mice, strain differences in MGS scores in animals not exposed to any painful interventions was demonstrated. Miller and Leach in 2015 [58] found that C3H/He mice showed significantly higher scores than CD-1 and C57BL/6 animals, although the order of effect for the latter two strains was different between males and females. In female BALB/c mice the grimace score was even higher than C3H/He (males were not investigated in this study). Cho et al. [65] in 2019 similarly demonstrated a difference in MGS scores post-craniotomy, with C57BL/6 mice with lower scores than CD-1 animals [65]. However, in pairwise comparisons of the CBA and DBA/2 strains in two further studies, no differences were found [66,67]. It was suggested by some authors that detection of facial features in dark animals may be more difficult [65,68]. Improving the image quality and providing a contrasting background color when recording appear to mitigate the effects [18], hence this may not be a feature of animal pigmentation per se. It should, however, be noted that in the Miller and Leach [58] 2015 study, female C57BL/6 animals were not scored the lowest; that place being taken by the white CD-1 animals. Brown C3H animals also occupied an intermediate position. In a clinical scenario where real-time scoring is likely to take place the issue of poor background contrast on videos is not of concern. However, some investigation of the effects of color on live grimace scoring is warranted since it may be equally as difficult for a human observer to distinguish features such as whiskers against a similar coat color background, especially when trying to observe at a distance so as not to influence the animal’s behavior.

Differences between sexes have also been uncovered in research to date on the MGS, but results are complex and suggest there may be strain interactions. For example, Miller and Leach [58] observed no differences in MGS scores between male and female C57BL/6 mice [58]. However in the same study, both CD-1 and C3H/He males had greater scores than their female counterparts [58]. Similarly, male BALB/c mice had higher grimace scores than females [69]. Alternately, Cho et al. [65] found no sex differences in CD-1 mice, although differences in response to analgesic were noted with females appearing to respond to carprofen with a reduction in grimace score more readily than males [65]. In rats, limited studies were carried out into sex differences but no differences were found in the original validation study [21], or in a later study [70]. Unfortunately, it appears that most grimace studies in rats and mice appear to have been conducted in one sex, with a large proportion using male animals, see e.g., [52,71,72,73]. This bias in study design toward males, coupled with the enhanced understanding of the existence of different pathways and immune-cell types for pain processing between male and female rodents [74], renders extrapolation of findings to female rodents problematic.

#### 4.2.2. Impact of Routine Procedures

It is clear that procedures occurring fairly often as part of vivarium routines may influence responses and should be taken into consideration when considering practical implementation of the grimace scales. For example, several studies evaluated the impact of anesthetics on rodent grimace scales. In general, both inhalational and injectable anesthetics lead to a short-term increase in grimace scores in both rats [73] and mice [66,75,76], although strain differences in the presence of this response were reported [66,75,76]. While this response is generally short-lived, repeated exposures lead to enhanced duration of the increase [68,73]. This is a particular consideration since grimace assessment would typically occur post-operatively to allow rescue analgesia administration and there is suggestion that the score increase may persist for up to a few hours post anesthesia [75,76].

There is a growing body of evidence that non-aversive handling of mice leads to reduced anxiety and improved resilience in the face of accompanying pain [77,78,79]. Cupping or tunnel handling are proposed as alternatives to the traditional method of picking up by the tail [78]. Perhaps somewhat surprisingly given the reported specificity of the MGS for pain there is some evidence that method of routine handling influences MGS with increased scores in mice handled by the tail compared to those that were tunnel handled [69]. This contradicts the findings of a previous study where no differences between the two methods were reported [67]. This is an area that should be a priority for further investigation for several reasons. Firstly, since non-aversive methods have not been widely incorporated into laboratory animal practice, especially among researchers [80], it is quite likely that mice even within one study will be subject to different handling techniques. Any effect of handling method on grimace score could therefore confound interpretation of grimace scores used to determine research protocol effects on pain. Secondly, while there appears to have been no dedicated study on whether tail handling induces pain, there is suggestion that it is non-painful, yet aversive [78]. If the method is actually non-painful this calls into question the specificity of the MGS for pain, and therefore whether it has construct validity.

Ear tagging or ear notching are routine handling procedures used to permanently identify laboratory animals [81]. These procedures are known to cause acute pain as reflected by alterations in physiological indices such as heart rate and blood pressure [82]. However, the results obtained by Miller and Leach [81] in mice did not reveal any change to MGS scores as a result of ear notching [81]. In a later mouse study, with a factorial study design evaluating handling method with ear tagging or tattooing, MGS increased following ear tagging but tattooing or restraint had no impact on scores [69]. Alternately, Keating et al. [22] in 2012 showed that ear tattooing in rabbits led to increases in rabbit grimace scale scores that were ameliorated by the application of a local topical anaesthetic (lidocaine/prilocaine) [22]. Corticosterone measures in this study suggest that the pain response was short-lived and had resolved by 1-h post-procedure. Given that only three studies, performed in different species, evaluated these common procedures, it would be unwise to draw firm conclusions. However, the lack of grimace score increase in the Miller and Leach [81] study does imply that the scale may not be sensitive to pain of a mild and short-lived nature either intrinsically, or as a result of practical features whereby the pain is missed due to the scoring process required. Conversely, this finding provides some evidence that routine procedures may have minimal effect on grimace scales, thus reducing potential confounding when using the scales for humane endpoint implementation. When reconciling the difference in findings between this [81], and the later study, Roughan and Sevenoaks [69] in 2019 speculated that ear tagging may be perceived as more painful than notching due to the prolonged irritation by the tag [69].

#### 4.2.3. Environmental Impacts

If the grimace scales are to be used as a practical tool they need to be repeatable across time and conditions, and not subject to extraneous influences. This requirement also relates to their face validity as reliable indicators of pain. In common with the other factors that may influence the scales, there has been limited research in this sphere.

Miller and Leach in 2015 [58] performed a comprehensive evaluation of some of the factors that might be predicted to influence grimace responses. One factor that may have an impact is the circadian cycle and whether differences in score occur across the day. For both live scoring and retrospective scoring, there were largely no differences seen between scores dependent on whether scoring took place in the morning, lunchtime or at the end of the day. There were some exceptions to this with BALB/c mice showing a greater live MGS score at noon compared to am and C57BL/6 mice showing higher retrospective MGS scores in both afternoon time points in comparison to the morning. It should be noted that in this, as in the majority of the studies examining grimace scores, animals were scored during the light phase of the circadian cycle when they would be expected to be inactive. There is some evidence that grimace scores may not be comparable between dark and light conditions with the finding that MGS was higher in the dark than in bright light in CD1 mice treated with a peptide believed to induce pain and migraine symptoms [83]. Analysis of the action units showed that the transition to light caused a significant decrease in orbital tightening and nose bulge [83]. Given that this finding was also observed in vehicle controls it appears unrelated to the migraine symptoms, and may be an aspect of normal biology needing consideration. Alternately, Matsumiya et al. 2012 found no difference in baseline MGS scores between morning and evening but did find that in operated animals scores were higher in the dark cycle, implying that pain was greater in the mice active phase [84]. However, in consideration of the use of the scales as a practical tool the reality is that most scoring will occur during the working day, in the light cycle, and therefore the findings of Miller and Leach in 2015 [58] provide confidence that time of scoring should not influence the score. A further important outcome from Miller and Leach [58] was that there was no effect of repeatedly being placed in the photography boxes on grimace score i.e., a habituation effect over the three occasions used [58]. The later study by Jirkof et al. [85] in 2020 supports this finding. This provides assurance that longitudinal monitoring post-procedure could occur throughout the day without the need to account for time of day or habituation to the box. However, as discussed earlier the need to remove animals to a separate box does impede the practical application of the test. Further study should consider time of day effects in the non-stimulated home environment.

There is further evidence of an impact of the external environment on grimace scores. Sorge et al. [59] compared grimace responses of mice and rats recorded after a painful insult, in the presence of a male compared to a female. Significant decreases in grimace response were recorded compared to the situation with no observer in the room. Females did not induce such a change. The findings therefore suggest that olfactory cues from human males lead to a physiological stress response, and associated stress-induced analgesia.

There is also some evidence of inter-laboratory variation in the outputs obtained from behavioral testing, to include MGS scores. In a multicenter study, Jirkof et al. [85] in 2020 demonstrated some quantitative differences in scores, although they were qualitatively comparable (direction of effect). However, variability between research centers in the MGS, especially when presented as a median score, was less pronounced than in burrowing behavior readouts [84]. This inter-lab variability was recognized across the spectrum of preclinical research pursuits, arising as a result of environmental variables leading to stress [86]. While this issue may be a concern when considering basic-to-clinical translation and reproducibility, it is less likely to be of concern for clinical application of the grimace scales. As a clinical tool, provided good inter and intra-observer, and thus intra-site agreement is obtained, grimace scores may be relied on locally for welfare determination subject to some of the other caveats discussed in this paper.

### 4.3. Validity

If grimace scales are to be implemented as a routine clinical assessment tool in biomedical research facilities, there needs to be a clear understanding of whether they are specific to pain, and can reliably measure pain in the models being used. This is important because it influences the animal caretaker’s decision as regard to treatment options, for example, whether analgesics will be effective in mitigating clinical signs. It can be seen from the above discussion that there are a range of external factors that affect grimace scores, speaking to their validity as a pain assessment tool; anesthetics are a prime example. Setting aside the lack of study into their application in a real-time scenario, which influences their generalizability, another key concern is whether they are valid for all pain types. Results of the original Langford et al. [18] in 2010 study suggested that the technique was only applicable for acute pain states [18], since changes were not recorded after the application of traditional models of chronic pain, such as chronic constriction injury (CCI). However, there have now been a range of studies, largely performed in mice, which suggest that the grimace scale may be applicable for pain that is chronic or neuropathic in nature, or of a non-surgical origin (see [36] for detailed discussion).

The study findings of Akintola et al. [52] in 2017 contradict the previous results of Langford et al. 2010 with both RGS and MGS increasing after application of the CCI model in these species. Pain arising from cancer has also been shown to cause an elevation of the MGS, for example in colo-rectal cancer [47] and in a metastatic breast cancer model [49,50]. The MGS was successfully used in models expected to produce pain of a neuropathic nature, for example in headache and migraine [55,87] and craniotomy [65]. There is also suggestion that pain of a visceral nature elevates scores based on studies evaluating colonic nociception [88], pelvic pain [89], colitis [61], and alimentary mucositis [62,63]. Hereditary sickle cell disease frequently leads to painful episodes in human patients. Cold treatment of transgenic sickle mice led to increased grimace scores, which were alleviated using a known analgesic agent. Furthermore, body changes of decreased length and increased back curvature were also correlated with the change in grimace scores [56]. These findings lend support to the proposition that the grimace scales have good construct validity for non-acute pain.

Despite these findings, results from other studies implies that further evaluation of the grimace techniques are necessary to ascertain validity. For example, in contradiction to later work [62,63] demonstrating elevations in scores in rats and mice with mucositis, Whittaker et al. [90] in 2015 found no change in grimace scores in a rat model, albeit using retrospective rather than real-time scoring. However, this study did find increases in frequency of established behavioral indicators of pain such as back arching and twitching [90]. Alternately, Leung et al. [61] in a rat DSS- colitis model found grimace score increases in the absence of an increase in composite behavioral score.

Other studies also raise questions of whether the grimace scales are truly unique to pain. Caecal ligation and puncture models are commonly used to study sepsis [91]. While sepsis is undoubtedly a painful condition based on human reports [92], there is also an overwhelming cytokine response causing sickness behavior. Studies to date on this model [51,93] have not teased apart the possible contribution of this sickness response to the facial expression changes. There is a study that lends support to this idea; the work of Yamamoto et al. in 2016 [94], while not employing the published rat grimace scale, provides evidence that nausea influences the eye action unit. Toxin administration, which might also be expected to cause dual symptoms of pain and sickness, similarly elevated the MGS [95]. Furthermore, analgesic administration was not always successful in reducing the scores implying an alternate cause of the facial action unit response. Finally, head injury may alter the animal’s ability to influence the facial action units via neural mechanisms and render grimace scores unreliable [44].

### 4.4. Automation of Techniques

One of the main current barriers to widespread clinical application of the grimace scales is the lack of understanding as to their validity and reliability when used for live scoring. However, as illustrated, there is now a wealth of literature on the validity and application of retrospective techniques using video or photo footage. In a clinical scenario these methods have limited application due to the time taken to extract the images, perform the scoring and potentially combine scores using statistical methods. However, there was some investigation of a range of technologies which minimize the time taken for various aspects of this process. At the simplest level use of freeware video to JPG converter software can reduce the time associated with manual searching and capture of images from recorded video footage by automating the capture process [48]. However, this still requires manual viewing of the selected images to obtain unobstructed head shots. Sotocinal et al. [21] in 2011 developed Rodent Face Finder^®^ which is able to detect rodent eyes and ears to generate stills of rodent faces. This software was used in a range of studies measuring grimace scores in both rats and mice see e.g., [44,52,84,96]. Recently, another research group generated an algorithm to generate repeatable, non-observer biased, standardized and randomized pictures in one step. The authors suggest that their system offers benefits in scoring animals with dark fur and allowing several animals to be filmed and generate images simultaneously [97]. They further went on to show that the system was robust across several facilities potentially minimizing issues around inter-laboratory variability as discussed previously [98].

This process of semi-automation makes grimace scoring somewhat more applicable to a clinical environment but the time taken to manually score images is still likely to be a barrier to implementation. In recent years, there has been some progress on further automation of facial expression recognition using machine learning techniques. Deep learning methods allow classification and predictions on the data without previous feature design [99]. Tuttle et al. 2018 [99] first trained a neural network using human scored mouse images. Their system was highly accurate (94% agreement with human scores) for a binary (pain versus no pain) output, with scores correlating highly with human-assigned scores. Other groups have similarly demonstrated the promise of deep learning methods for use with the MGS when based on binary outputs [100,101]. Progress has also been made toward automating a facial pain expression system in sheep using techniques used in human facial recognition [102,103].

These automation methods are in their infancy and no doubt there will be further development of these techniques over the next few years. A key issue currently is that they lack sensitivity- being only able to distinguish a painful from a non-painful state. This renders their current use for welfare assessment and endpoint implementation limited. However, given the success and practical implementation of machine learning methods in recognition of human facial expression, it is likely to be only a matter of time before a similar level of sensitivity of scoring will be possible in animal–focused methods [104].

## 5. Practical Considerations

The above discussion highlights some areas in need for future research particularly in regard to practical usage of the grimace scales in laboratory animal medicine. A key issue is what to do with the data when it is acquired, and what it means for the animal. In research use of the grimace scales, statistically significant differences in grimace scores in comparison with controls are typically reported. However, in a clinical scenario, a mass of data or control animals’ results may not be available to make this comparison on the spot. Moreover, statistical significance may not always equate with clinical significance. There needs to be ascertainment of the level of grimace score at which pain is actually occurring, since the evidence suggests that grimace scores in healthy animals are rarely zero [58]. Some attempts were made to address this issue with the development of intervention thresholds. Scores that are above this level signify that the animal is in pain, and consideration should be given to providing rescue analgesia [105]. These thresholds would need to be derived based on the method of combining individual action unit scores used, for example in the MGS summation of scores leads to a maximum of 10, whereas averaging leads to a maximum of 2. Oliver et al. [105] in 2014 determined for rats that 0.67/2 was a suitable intervention threshold. An intervention threshold has also been suggested for sheep (above 5/10) [25], and cats (0.39/1) [27]. It was considered by the authors in the sheep study that false positives for pain were unlikely above this cut-off score, although it was acknowledged that due to low test sensitivity some animals scoring below five may have a painful condition [25]. Since individuals experience pain differently, and there are associated sex differences in both pain experience and response to analgesics, work is needed to tailor intervention thresholds considering these factors. Additionally, consideration should be given to the fluctuating nature of pain [106], rendering regular monitoring, scoring and comparison with previous scores critical [44]. Monitoring staff need to consider tailoring of analgesic regimes due to animals potentially being in more pain in their active phase (see e.g., [84]), which may fall outside of staffed hours.

Given, the lack of established intervention thresholds perhaps the best current advice would be to use a holistic approach in pain assessment and consider grimace scores alongside other measures of well-being such as standard clinical scoring, and where possible look for trends in score progression within the same animal to guide decision-making. Animal carers also need to consider the potential impacts of inter and intra-observer variability on scoring which may be significant when statistical methods on group data are not used to smooth out variability. A prudent approach, where possible, would be to use the same scorer in a clinical case. This concern also brings up the issue of training of scorers which has received minimal research attention. Some studies implied that minimal training, such as the provision of online instructions, is all that is necessary to achieve consistent results between expert and novice scorers [69,107]. However, another study has shown that more in-depth training, using practice scoring associated with structured opportunities for discussion, enhanced scoring ability [108].

## 6. Conclusions and Future Directions

Despite 10 years of investigation, widespread uptake of grimace scoring in biomedical research has not occurred. The grimace scales offer enormous potential for clinical use in biomedical research. They are simple, require no equipment and were shown through research study to have good construct validity for most conditions. However, the methodology used in research on grimace scales is unlikely to lend to practical implementation due to its time intensive and retrospective nature. To date, few studies have investigated the validity of grimace scales in scenarios requiring on the spot pain assessment and clinical decision-making. Key areas for focus are on grimace score validity in animals housed in home cages, the reliability of using a limited number of real-time observation points, the impact of observers on scores, and the need for observer training. This is an area in urgent need for future research to realize the potential value of grimace scales.

One area that has received attention is the automation of scales using machine learning and algorithmic methods. This is a welcome development and will enhance the practical potential of grimace scales. It is hoped that in future years, grimace scale scoring may just be one of several outcome measures acquired routinely through facility-automated systems. This scenario is most likely to address the practical issues inherent when dealing with large numbers of animals, going some way toward addressing public concern around ethical decision-making in biomedical research.

## Figures and Tables

**Table 1:** Original studies in which grimace scales were developed for a range of species commonly used as laboratory animals.

Species	Validation Method	Action Units	Study
Mouse Grimace Scale (MGS)	Fourteen commonly used preclinical pain assays.	Five Units: (1) Orbital tightening, (2) Nose bulge, (3) Cheek bulge, (4) Ear position and (5) Whisker change	[18]
Rat Grimace Scale (RGS)	Three pain-eliciting procedures performed. (1) intraplantar administration of Complete Freund’s adjuvant (CFA); (2) intra-articular administration of kaolin/carrageenan; and (3) post-operative pain after laparotomy.	Four Units: (1) Orbital tightening, (2) Nose/cheek flattening, (3) Ear changes, (4) Whisker change	[21]
Rabbit Grimace Scale (RbtGS)	Pain caused by ear tattooing, a routine procedure used to identify rabbits. Analgesic test applied in the form of prilocaine/lidocaine (EMLA) local anesthetic	Five Units: (1) Orbital tightening, (2) Cheek flattening, (3) Nose shape, (4) Whisker position, (5) Ear position.	[22]
Sheep Grimace Scales (Sheep Pain Facial Expression Scale—SPFES)	Clinical model based on mastitis and footrot	Five Units: (1) Orbital tightening, (2) Cheek tightness, (3) Ear position, (4) Lip and jaw profile, (5) Nostril and philtrum position	[25]
Ferret (FGS)	Surgery involving the implantation of an intraperitoneal telemetry catheter	Five Units: (1) Orbital tightening, (2) Nose bulging, (3) Cheek bulging, (4) ear changes, (5) Whisker retraction	[26]
Piglets (PGS)	Castration and tail docking. Validated orbital tightening for tail docking but remarked that further validation needed.	Ten used for development, later study [37] modified to three: (1) Ear Position, (2) Cheek Tightening/Nose bulge, (3) Orbital Tightening	[37,38]
Cat (FGS)	Acute pain arising as a result of a variety of clinical conditions	Five units: (1) Ear position, (2) Orbital tightening, (3) Muzzle tension, (4) Whisker change, (5) Head position	[27]
Horse (HGS)	Surgical castration	Six units: (1) Stiffly backward ears, (2) Orbital tightening, (3) Tension above the eye area, (4) Prominent strained chewing muscles, (5) Mouth strained and pronounced chin, (6) Strained nostrils and flattening of the profile	[28]

## References

[1] Mota-Rojas D., Velarde A., Maris-Huertas S., Cajiao M.N. (2016). Animal Welfare, A Global Vision in Ibero-America.

[2] Lewejohann L., Schwabe K., Häger C., Jirkof P. (2020). Impulse for animal welfare outside the experiment. Lab. Anim..

[3] Taylor K., Gordon N., Langley G., Higgins W. (2008). Estimates for worldwide laboratory animal use in 2005. Altern. Lab. Anim..

[4] Mota-Rojas D., Orihuela A., Martínez-Burnes J., Gómez J., Mora-Medina P., Alavez B., Ramírez L., González-Lozano M. (2020). Neurological modulation of facial expressions in pigs and implications for production. J. Anim. Behav. Biometeorol..

[5] Mota-Rojas D., Olmos-Hernández A., Verduzco-Mendoza A., Lecona-Butrón H., Martínez-Burnes J., Mora-Medina P., Gómez-Prado J., Orihuela A. (2020). Infrared thermal imaging associated with pain in laboratory animals. Exp. Anim..

[6] Baumans V. (2005). Science-based assessment of animal welfare: Laboratory animals. Rev. Sci. Tech. OIE.

[7] Lezama-García K., Orihuela A., Olmos-Hernández A., Reyes-Long S., Mota-Rojas D. (2019). Facial expressions and emotions in domestic animals. CAB Rev. Perspect. Agric. Vet. Sci. Nutr. Nat. Resour..

[8] Finlayson K., Lampe J., Hintze S., Würbel H., Melotti L. (2016). Facial indicators of positive emotions in rats. PLoS ONE.

[9] Whittaker A. (2019). The role of behavioural assessment in determining ‘positive’ affective states in animals. CAB Rev. Perspect. Agric. Vet. Sci. Nutr. Nat. Resour..

[10] Boissy A., Manteuffel G., Jensen M.B., Moe R.O., Spruijt B., Keeling L.J., Winckler C., Forkman B., Dimitrov I., Langbein J. (2007). Assessment of positive emotions in animals to improve their welfare. Physiol. Behav..

[11] Panksepp J. (2005). Affective consciousness: Core emotional feelings in animals and humans. Conscious. Cogn..

[12] Carbone L., Austin J. (2016). Pain and laboratory animals: Publication practices for better data reproducibility and better animal welfare. PLoS ONE.

[13] Peterson N.C., Nunamaker E.A., Turner P.V. (2017). To treat or not to treat: The effects of pain on experimental parameters. Comp. Med..

[14] Zurlo J., Hutchinson E. (2014). Refinement. ALTEX.

[15] Bennett V., Gourkow N., Mills D. (2017). Facial correlates of emotional behaviour in the domestic cat (Felis catus). Behav. Process..

[16] Ekman P. (1992). Are there basic emotions?. Psychol. Rev..

[17] Mota-Rojas D., Orihuela A., Strappini-Asteggiano A., Cajiao-Pachón M.N., Agüera-Buendía E., Mora-Medina P., Ghezzi M., Alonso-Spilsbury M. (2018). Teaching animal welfare in veterinary schools in Latin America. Int. J. Vet. Sci. Med..

[18] Langford D.J., Bailey A.L., Chanda M.L., Clarke S.E., Drummond T.E., Echols S., Glick S., Ingrao J., Klassen-Ross T., LaCroix-Fralish M.L. (2010). Coding of facial expressions of pain in the laboratory mouse. Nat. Methods.

[19] Langford D.J. (2006). Social modulation of pain as evidence for empathy in mice. Science.

[20] Dolensek N., Gehrlach D.A., Klein A.S., Gogolla N. (2020). Facial expressions of emotion states and their neuronal correlates in mice. Science.

[21] Sotocinal S.G., Sorge R.E., Zaloum A., Tuttle A.H., Martin L.J., Wieskopf J.S., Mapplebeck J.C.S., Wei P., Zhan S., Zhang S. (2011). The Rat Grimace Scale: A partially automated method for quantifying pain in the laboratory rat via facial expressions. Mol. Pain.

[22] Keating S.C.J., Thomas A.A., Flecknell P.A., Leach M.C. (2012). Evaluation of EMLA cream for preventing pain during tattooing of rabbits: Changes in physiological, behavioural and facial expression responses. PLoS ONE.

[23] Hampshire V., Robertson S. (2015). Using the facial grimace scale to evaluate rabbit wellness in post-procedural monitoring. Lab. Anim..

[24] Häger C., Biernot S., Buettner M., Glage S., Keubler L.M., Held N., Bleich E.M., Otto K., Müller C.W., Decker S. (2017). The Sheep Grimace Scale as an indicator of post-operative distress and pain in laboratory sheep. PLoS ONE.

[25] McLennan K.M., Rebelo C.J., Corke M.J., Holmes M.A., Leach M.C., Constantino-Casas F. (2016). Development of a facial expression scale using footrot and mastitis as models of pain in sheep. Appl. Anim. Behav. Sci..

[26] Reijgwart M.L., Schoemaker N.J., Pascuzzo R., Leach M.C., Stodel M., De Nies L., Hendriksen C.F.M., Van Der Meer M., Vinke C.M., Van Zeeland Y.R.A. (2017). The composition and initial evaluation of a grimace scale in ferrets after surgical implantation of a telemetry probe. PLoS ONE.

[27] Evangelista M.C., Watanabe R., Leung V.S.Y., Monteiro B.P., O’Toole E., Pang D.S.J., Steagall P.V. (2019). Facial expressions of pain in cats: The development and validation of a Feline Grimace Scale. Sci. Rep..

[28] Costa E.D., Minero M., Lebelt D., Stucke D., Canali E., Leach M.C. (2014). Development of the horse grimace scale (HGS) as a pain assessment tool in horses undergoing routine castration. PLoS ONE.

[29] Apkarian A.V., Hashmi J.A., Baliki M.N. (2011). Pain and the brain: Specificity and plasticity of the brain in clinical chronic pain. Pain.

[30] Blackburn-Munro G. (2004). Pain-like behaviours in animals—How human are they?. Trends Pharmacol. Sci..

[31] Nagakura Y. (2016). The need for fundamental reforms in the pain research field to develop innovative drugs. Expert Opin. Drug Discov..

[32] Nagakura Y., Miwa M., Yoshida M., Miura R., Tanei S., Tsuji M., Takeda H. (2019). Spontaneous pain-associated facial expression and efficacy of clinically used drugs in the reserpine-induced rat model of fibromyalgia. Eur. J. Pharmacol..

[33] Serizawa K., Tomizawa-Shinohara H., Yasuno H., Yogo K., Matsumoto Y. (2019). Anti-IL-6 receptor antibody inhibits spontaneous pain at the pre-onset of experimental autoimmune encephalomyelitis in Mice. Front. Neurol..

[34] LeResche L. (1982). Facial expression in pain: A study of candid photographs. J. Nonverbal Behav..

[35] Williams A.C.D.C. (2002). Facial expression of pain: An evolutionary account. Behav. Brain Sci..

[36] Mogil J.S., Pang D.S., Dutra G.G.S., Chambers C.T. (2020). The development and use of facial grimace scales for pain measurement in animals. Neurosci. Biobehav. Rev..

[37] Viscardi A.V., Hunniford M., Lawlis P., Leach M., Turner P.V. (2017). Development of a piglet grimace scale to evaluate piglet pain using facial expressions following castration and tail docking: A pilot study. Front. Vet. Sci..

[38] Di Giminiani P., Brierley V.L., Scollo A., Gottardo F., Malcolm E.M., Edwards S.A., Leach M.C. (2016). The assessment of facial expressions in piglets undergoing tail docking and castration: Toward the development of the piglet grimace scale. Front. Vet. Sci..

[39] Bateson P. (1991). Assessment of pain in animals. Anim. Behav..

[40] Whittaker A.L., Howarth G.S. (2014). Use of spontaneous behaviour measures to assess pain in laboratory rats and mice: How are we progressing?. Appl. Anim. Behav. Sci..

[41] Rutherford K. (2002). Assessing pain in animals. Anim. Welf..

[42] De C Williams A.C. (2019). Persistence of pain in humans and other mammals. Philos. Trans. R. Soc. B Biol. Sci..

[43] Walters E.T., De C Williams A.C. (2019). Evolution of mechanisms and behaviour important for pain. Philos. Trans. R. Soc. B Biol. Sci..

[44] McLennan K.M., Miller A.L., Costa E.D., Stucke D., Corke M.J., Broom D.M., Leach M.C. (2019). Conceptual and methodological issues relating to pain assessment in mammals: The development and utilisation of pain facial expression scales. Appl. Anim. Behav. Sci..

[45] Bendinger T., Plunkett N. (2016). Measurement in pain medicine. BJA Educ..

[46] Good M., Stiller C., Zauszniewski J.A., Anderson G.C., Stanton-Hicks M., Grass J.A. (2001). Sensation and distress of pain scales: Reliability, validity, and sensitivity. J. Nurs. Meas..

[47] Chartier L.C., Hebart M.L., Howarth G.S., Whittaker A.L., Mashtoub S. (2020). Affective state determination in a mouse model of colitis-associated colorectal cancer. PLoS ONE.

[48] George R.P., Howarth G.S., Whittaker A.L. (2019). Use of the rat grimace scale to evaluate visceral pain in a model of chemotherapy-induced mucositis. Animals.

[49] De Almeida A.S., Rigo F.K., De Prá S.D.T., Milioli A.M., Dalenogare D.P., Pereira G.C., Ritter C.D.S., Peres D.S., Antoniazzi C.T.D., Stein C. (2019). Characterization of cancer-induced nociception in a murine model of breast carcinoma. Cell. Mol. Neurobiol..

[50] De Almeida A.S., Rigo F.K., De Prá S.D.T., Milioli A.M., Pereira G.C., Lückemeyer D.D., Antoniazzi C.T., Kudsi S.Q., Araújo D., Oliveira S.M. (2020). Role of transient receptor potential ankyrin 1 (TRPA1) on nociception caused by a murine model of breast carcinoma. Pharmacol. Res..

[51] Mai S.H.C., Sharma N., Kwong A.C., Dwivedi D.J., Khan M., Grin P., Fox-Robichaud A.E., Liaw P.C. (2018). Body temperature and mouse scoring systems as surrogate markers of death in cecal ligation and puncture sepsis. Intensiv. Care Med. Exp..

[52] Akintola T., Raver C., Studlack P., Uddin O., Masri R., Keller A. (2017). The grimace scale reliably assesses chronic pain in a rodent model of trigeminal neuropathic pain. Neurobiol. Pain.

[53] Akintola T., Tricou C., Raver C., Castro A., Colloca L., Keller A. (2019). In search of a rodent model of placebo analgesia in chronic orofacial neuropathic pain. Neurobiol. Pain.

[54] Duffy S.S., Perera C.J., Makker P.G.S., Lees J.G., Carrive P., Moalem-Taylor G. (2016). Peripheral and central neuroinflammatory changes and pain behaviors in an animal model of multiple sclerosis. Front. Immunol..

[55] Hassler S.N., Ahmad F.B., Burgos-Vega C.C., Boitano S., Vágner J., Price T.J., Dussor G. (2018). Protease activated receptor 2 (PAR2) activation causes migraine-like pain behaviors in mice. Cephalalgia.

[56] Mittal A., Gupta M., Lamarre Y., Jahagirdar B., Gupta K. (2016). Quantification of pain in sickle mice using facial expressions and body measurements. Blood Cells Mol. Dis..

[57] Gao M., Long H., Ma W., Liao L., Yang X., Zhou Y., Shan D., Huang R., Jian F., Wang Y. (2015). The role of periodontal ASIC3 in orofacial pain induced by experimental tooth movement in rats. Eur. J. Orthod..

[58] Miller A.L., Leach M.C. (2015). The mouse grimace scale: A clinically useful tool?. PLoS ONE.

[59] Sorge R.E., Martin L.J., Isbester K.A., Sotocinal S.G., Rosen S., Tuttle A.H., Wieskopf J.S., Acland E.L., Dokova A., Kadoura B. (2014). Olfactory exposure to males, including men, causes stress and related analgesia in rodents. Nat. Methods.

[60] Leung V., Zhang E., Pang D.S. (2016). Real-time application of the Rat Grimace Scale as a welfare refinement in laboratory rats. Sci. Rep..

[61] Leung V.S., Benoit-Biancamano M.O., Pang D.S. (2019). Performance of behavioral assays: The Rat Grimace Scale, burrowing activity and a composite behavior score to identify visceral pain in an acute and chronic colitis model. PAIN Rep..

[62] Wardill H.R., Gibson R.J., Van Sebille Y.Z., Secombe K.R., Coller J.K., White I.A., Manavis J., Hutchinson M.R., Staikopoulos V., Logan R. (2016). Irinotecan-induced gastrointestinal dysfunction and pain are mediated by common TLR4-dependent mechanisms. Mol. Cancer Ther..

[63] Gibson R.J., Coller J.K., Wardill H.R., Hutchinson M.R., Smid S., Bowen J.M. (2015). Chemotherapy-induced gut toxicity and pain: Involvement of TLRs. Support. Care Cancer.

[64] Hsi Z.Y., Stewart L.A., Lloyd K.C.K., Grimsrud K.N. (2020). Hypoglycemia after bariatric surgery in mice and optimal dosage and efficacy of glucose supplementation. Comp. Med..

[65] Cho C., Michalidis V., Lecker I., Collymore C., Hanwell D., Loka M., Danesh M., Pham C., Urban P., Bonin R.P. (2019). Evaluating analgesic efficacy and administration route following craniotomy in mice using the grimace scale. Sci. Rep..

[66] Miller A.L., Kitson G., Skalkoyannis B., Leach M. (2015). The effect of isoflurane anaesthesia and buprenorphine on the mouse grimace scale and behaviour in CBA and DBA/2 mice. Appl. Anim. Behav. Sci..

[67] Miller A.L., Leach M.C. (2016). The effect of handling method on the mouse grimace scale in two strains of laboratory mice. Lab. Anim..

[68] Costa E.D., Pascuzzo R., Leach M.C., Dai F., Lebelt D., Vantini S., Minero M. (2018). Can grimace scales estimate the pain status in horses and mice? A statistical approach to identify a classifier. PLoS ONE.

[69] Roughan J.V., Sevenoaks T. (2019). Welfare and scientific considerations of tattooing and ear tagging for mouse identification. J. Am. Assoc. Lab. Anim. Sci..

[70] Waite M.E., Tomkovich A., Quinn T.L., Schumann A.P., Dewberry L.S., Totsch S.K., Sorge R.E. (2015). Efficacy of common analgesics for postsurgical pain in rats. J. Am. Assoc. Lab. Anim. Sci..

[71] Wang S., Kim M., Ali Z., Ong K., Pae E.K., Chung M.K. (2019). Trpv1 and trpv1-expressing nociceptors mediate orofacial pain behaviors in a mouse model of orthodontic tooth movement. Front Physiol.

[72] Zhu Y., Wang S., Long H., Zhu J., Jian F., Ye N., Lai W. (2016). Effect of static magnetic field on pain level and expression of P2X3 receptors in the trigeminal ganglion in mice following experimental tooth movement. Bioelectromagnetics.

[73] Miller A.L., Golledge H.D.R., Leach M.C. (2016). The influence of isoflurane anaesthesia on the rat grimace scale. PLoS ONE.

[74] Sorge R.E., Mapplebeck J.C.S., Rosen S., Beggs S., Taves S., Alexander J.K., Martin L.J., Austin J.-S., Sotocinal S.G., Chen D. (2015). Different immune cells mediate mechanical pain hypersensitivity in male and female mice. Nat. Neurosci..

[75] Hohlbaum K., Bert B., Dietze S., Palme R., Fink H., Thöne-Reineke C. (2017). Severity classification of repeated isoflurane anesthesia in C57BL/6JRj mice—Assessing the degree of distress. PLoS ONE.

[76] Hohlbaum K., Bert B., Dietze S., Palme R., Fink H., Thöne-Reineke C. (2018). Impact of repeated anesthesia with ketamine and xylazine on the well-being of C57BL/6JRj mice. PLoS ONE.

[77] Gouveia K., Hurst J.L. (2017). Optimising reliability of mouse performance in behavioural testing: The major role of non-aversive handling. Sci. Rep..

[78] Hurst J.L., West R.S. (2010). Taming anxiety in laboratory mice. Nat. Methods.

[79] Gouveia K., Hurst J.L. (2013). Reducing mouse anxiety during handling: Effect of experience with handling tunnels. PLoS ONE.

[80] Henderson L.J., Smulders T.V., Roughan J.V. (2020). Identifying obstacles preventing the uptake of tunnel handling methods for laboratory mice: An international thematic survey. PLoS ONE.

[81] Miller A.L., Leach M. (2014). Using the mouse grimace scale to assess pain associated with routine ear notching and the effect of analgesia in laboratory mice. Lab. Anim..

[82] Kasanen I.H.E., Voipio H.-M., Leskinen H., Luodonpää M., Nevalainen T.O. (2011). Comparison of ear tattoo, ear notching and microtattoo in rats undergoing cardiovascular telemetry. Lab. Anim..

[83] Rea B.J., Wattiez A.-S., Waite J.S., Castonguay W.C., Schmidt C.M., Fairbanks A.M., Robertson B.R., Brown C.J., Mason B.N., Moldovan-Loomis M.-C. (2018). Peripherally administered calcitonin gene–related peptide induces spontaneous pain in mice. Pain.

[84] Matsumiya L.C., Sorge R.E., Sotocinal S.G., Tabaka J.M., Wieskopf J.S., Zaloum A., King O.D., Mogil J.S. (2012). Using the mouse grimace scale to reevaluate the efficacy of postoperative analgesics in laboratory mice. J. Am. Assoc. Lab. Anim. Sci..

[85] Jirkof P., Abdelrahman A., Bleich A., Durst M., Keubler L.M., Potschka H., Struve B., Talbot S.R., Vollmar B., Zechner D. (2019). A safe bet? Inter-laboratory variability in behaviour-based severity assessment. Lab. Anim..

[86] Mogil J.S. (2017). Laboratory environmental factors and pain behavior: The relevance of unknown unknowns to reproducibility and translation. Lab. Anim..

[87] Burgos-Vega C.C., Quigley L.D., Dos Santos G.T., Yan F., Asiedu M., Jacobs B., Motina M., Safdar N., Yousuf H., Avona A. (2018). Non-invasive dural stimulation in mice: A novel preclinical model of migraine. Cephalalgia.

[88] Hassan A.M., Jain P., Mayerhofer R., Fröhlich E.E., Farzi A., Reichmann F., Herzog H., Holzer P. (2017). Visceral hyperalgesia caused by peptide YY deletion and Y2 receptor antagonism. Sci. Rep..

[89] Bu X., Liu Y., Lu Q., Jin Z. (2015). Effects of “Danzhi Decoction” on chronic pelvic pain, hemodynamics, and proinflammatory factors in the murine model of sequelae of pelvic inflammatory disease. Evid. Based Complement. Altern. Med..

[90] Whittaker A.L., Leach M.C., Preston F.L., Lymn K.A., Howarth G.S. (2015). Effects of acute chemotherapy-induced mucositis on spontaneous behaviour and the grimace scale in laboratory rats. Lab. Anim..

[91] Toscano M.G., Ganea I., Gamero A.M. (2011). Cecal ligation puncture procedure. J. Vis. Exp..

[92] Nguyen H.B., Rivers E.P., Abrahamian F.M., Moran G.J., Abraham E., Trzeciak S., Huang D.T., Osborn T.M., Stevens D., Talan D.A. (2006). Severe sepsis and septic shock: Review of the literature and emergency department management guidelines. Ann. Emerg. Med..

[93] Dwivedi D.J., Grin P., Khan M., Prat A., Zhou J., Fox-Robichaud A.E., Seidah N.G., Liaw P.C. (2016). Differential expression of PCSK9 modulates infection, inflammation, and coagulation in a murine model of sepsis. Shock.

[94] Yamamoto K., Tatsutani S., Ishida T. (2017). Detection of nausea-like response in rats by monitoring facial expression. Front. Pharmacol..

[95] Herrera C., Bolton F., Arias A., Harrison R.A., Gutiérrez J.M. (2018). Analgesic effect of morphine and tramadol in standard toxicity assays in mice injected with venom of the snake Bothrops asper. Toxicon.

[96] Wong S.M., Tan S.J.X., Koh J., Zainul M., Phang G.S.S., Toh A., Babu K.R., Chooi K.F. The Rat Face Finder and Improved Assessment of Visceral Pain. Proceedings of the 9th SALAS Annual Regional Conference—Neuroscience: A New Frontier.

[97] Ernst L., Kopaczka M., Schulz M., Talbot S.R., Zieglowski L., Meyer M., Bruch S., Merhof D., Tolba R.H. (2019). Improvement of the Mouse Grimace Scale set-up for implementing a semi-automated Mouse Grimace Scale scoring (Part 1). Lab. Anim..

[98] Ernst L., Kopaczka M., Schulz M., Talbot S.R., Struve B., Häger C., Bleich A., Durst M., Jirkof P., Arras M. (2019). Semi-automated generation of pictures for the Mouse Grimace Scale: A multi-laboratory analysis (Part 2). Lab. Anim..

[99] Tuttle A.H., Molinaro M.J., Jethwa J.F., Sotocinal S.G., Prieto J.C., Styner M.A., Mogil J.S., Zylka M.J. (2018). A deep neural network to assess spontaneous pain from mouse facial expressions. Mol. Pain.

[100] Andresen N., Wöllhaf M., Hohlbaum K., Lewejohann L., Hellwich O., Thöne-Reineke C., Belik V. (2020). Towards a fully automated surveillance of well-being status in laboratory mice using deep learning: Starting with facial expression analysis. PLoS ONE.

[101] Eral M., Aktas C.C., Kocak E.E., Dalkara T., Halici U. Assessment of pain in mouse facial images. Proceedings of the 2016 20th National Biomedical Engineering Meeting (BIYOMUT).

[102] Mahmoud M., Lu Y., Hou X., McLennan K., Robinson P. (2018). Estimation of Pain in Sheep Using Computer Vision. Handbook of Pain and Palliative Care.

[103] McLennan K.M., Mahmoud M. (2019). Development of an automated pain facial expression detection system for sheep (ovis aries). Animals.

[104] Bartlett M., Littlewort G., Frank M., Lainscsek C., Fasel I., Movellan J. Recognizing Facial Expression: Machine Learning and Application to Spontaneous Behavior. Proceedings of the 2005 IEEE Computer Society Conference on Computer Vision and Pattern Recognition (CVPR’05)—Workshops.

[105] Oliver V., De Rantere D., Ritchie R., Chisholm J., Hecker K.G., Pang D.S. (2014). Psychometric assessment of the rat grimace scale and development of an analgesic intervention score. PLoS ONE.

[106] Baliki M.N., Chialvo D.R., Geha P.Y., Levy R.M., Harden R.N., Parrish T.B., Apkarian A.V. (2006). Chronic pain and the emotional brain: Specific brain activity associated with spontaneous fluctuations of intensity of chronic back pain. J. Neurosci..

[107] Roughan J.V., Bertrand H.G., Isles H.M. (2015). Meloxicam prevents COX-2-mediated post-surgical inflammation but not pain following laparotomy in mice. Eur. J. Pain.

[108] Zhang E.Q., Leung V.S., Pang D.S. (2019). Influence of rater training on inter- and intrarater reliability when using the rat grimace scale. J. Am. Assoc. Lab. Anim. Sci..

